# A cross-sectional analytical study assessing apoptotic index as a marker of disease progression in cervical dysplasia

**DOI:** 10.11604/pamj.2026.53.81.46693

**Published:** 2026-02-13

**Authors:** Trupti Amol Dongre, Pragati Jayant Karmarkar, Vidula Prashant Gowardhan, Shilpa Hajare

**Affiliations:** 1 Department of Pathology, NKP Salve Institute of Medical Sciences and Research Centre and Lata Mangeshkar Hospital, Nagpur, Maharashtra, India,; 2 Department of Community Medicine, NKP Salve Institute of Medical Sciences and Research Centre and Lata Mangeshkar Hospital, Nagpur, Maharashtra, India

**Keywords:** Apoptosis, apoptotic index, cervical epithelial dysplasia, cervical malignancy

## Abstract

**Introduction:**

cervical cancer is a major malignancy in women. In cancer cells, there is an equilibrium between cell proliferation and apoptosis. Defects in this pathway allow cells to replicate. Therefore, the apoptotic index (AI) can be used to assess dysplasia. The aim of this study was to assess the apoptotic index of various grades of cervical epithelial dysplasia.

**Methods:**

slides were retrieved from women with gynaecological complaints undergoing cervical biopsy or hysterectomy at a tertiary care hospital. The histopathology slides of 140 cases were studied from March 2021 to Feb 2022. Statistical analysis: we used SocSciStatistics for calculations. The slides were examined using Olympus microscope CH 20i at 40X magnification. Five fields were selected from each slide and the number of epithelial cells was counted. The apoptotic bodies were counted and evaluated. Apoptotic index was calculated as the number of apoptotic bodies/the total number of cells in one field, which was expressed as percentage (%).

**Results:**

a significant difference was observed between the mild and severe dysplasia groups. In squamous metaplasia, the mean AI was 1.03, while in mild cervical dysplasia it was 1.51. For moderate cervical dysplasia, the mean AI was 3.06, and for severe dysplasia, the mean AI was 5.23. In cases with overtly malignant cervical lesions, the mean AI was 7.35.

**Conclusion:**

this study confirmed the clinical significance of apoptosis in evaluating the course of cervical epithelial dysplasia, which can also serve as a prognostic indicator for premalignant and malignant cervical lesions.

## Introduction

According to the World Health Organization, cervical cancer is the fourth most common cancer in women worldwide. Cervical dysplasia is a precursor to cervical cancer. Cervical dysplasia is a condition in which healthy cells in the cervix undergo abnormal changes. Abnormal cells are not cancerous but can develop into cancer if they are not caught early and treated. Neoplastic growth is defined as cell proliferation minus cell loss. Cell loss can occur because of cell death, either by necrosis or apoptosis. Apoptosis has gained immense importance in the field of tumor biology. Apoptosis is a form of cell death in which a programmed sequence of events leads to the elimination of cells without the release of harmful substances. Apoptosis plays an important role, as it also eliminates potentially cancerous and virus infected cells like human papillomavirus (HPV) and maintains balance in the body. Human papillomavirus is a high-risk cause of cervical cancer. Dysregulation of the apoptotic pathway can either cause excessive removal or prolonged survival of the cell and provide more time for the accumulation of mutations, which can increase invasiveness during tumor progression, stimulate angiogenesis, deregulate cell proliferation, and interfere with differentiation [1]. Altered apoptotic pathways may lead to malignant transformation and tumor proliferation. Apoptotic bodies are characterized by nuclear condensation, cell shrinkage, membrane blebbing, and DNA fragmentation. Higher AI indicates high epithelial dysplasia. Objective: i) to calculate the apoptotic index in various grades of cervical epithelial dysplasia; ii) to compare the AI between various grades of epithelial dysplasia; iii) to predict the biological behaviour of cervical epithelial dysplasia based on AI.

## Methods

**Study design:** a cross-sectional analytical study was conducted to assess apoptotic index as a marker of disease progression in cervical dysplasia.

**Study setting and population:** the apoptotic index (AI) was measured on paraffin-embedded hematoxylin and eosin-stained slides from the diagnosed cases of cervical dysplasia of 140 cases. AI values were compared with the histological grades of cervical intraepithelial neoplasia (CIN) and invasive cervical carcinoma of patients registered in a tertiary care hospital.

### Data resource and measurement

**Data collection tool:** paraffin-embedded hematoxylin and eosin-stained slides from the diagnosed cases of cervical dysplasia of 140 cases.

**Data collection:** histopathology slides of 140 cases were studied and apoptotic index was calculated and entered in excel sheet. AI values were compared with the histological grades of CIN and invasive cervical carcinoma.

**Sample size:** histopathological slides of all patients having abnormal uterine bleeding or gynecological complaints who underwent cervical biopsy or hysterectomy were studied between March 21 and February 22. Inadequate material slides and slides with artifacts were excluded from the study.

**Data analysis:** the slides were examined using Olympus microscope CH 20i at 40X magnification. Five fields were selected from each slide and the number of epithelial cells was counted. The apoptotic bodies were counted and evaluated. Apoptotic index was calculated as the number of apoptotic bodies/total number of cells in one field, which was expressed as percentage (%). In this study, all samples were examined by two independent observers to minimize observer bias. Apoptotic index counts were performed separately for each observer. Under a light microscope, apoptotic bodies appear as round or oval masses of densely eosinophilic cytoplasm, containing fragments of condensed nuclear chromatin. These apoptotic bodies reflect the morphological changes that occur at various stages of the cell cycle. In cervical epithelial mild to moderate dysplasia, apoptotic bodies are mostly located in the basal layer. SocSciStatistics was used for calculations.

**Ethical consideration:** the study was approved by the institutional ethics committee, NKP Salve Institute of Medical Sciences and Research Centre and Lata Mangeshkar Hospital /Pharmacology/02/2021. Dated-27/02/2021.

## Results

**Figure 1:**
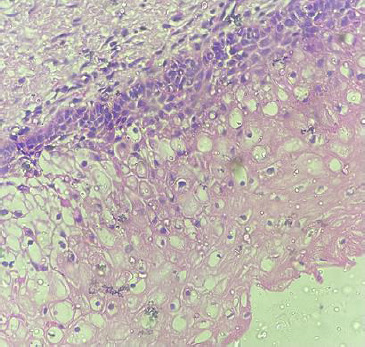
H&E; x 400, mild dysplasia in ectocervix

**Figure 2:**
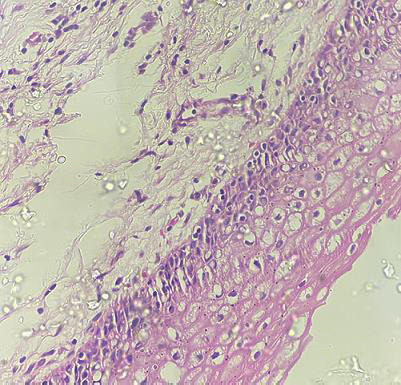
H&E; x 100, moderate dysplasia in ectocervix

**Figure 3:**
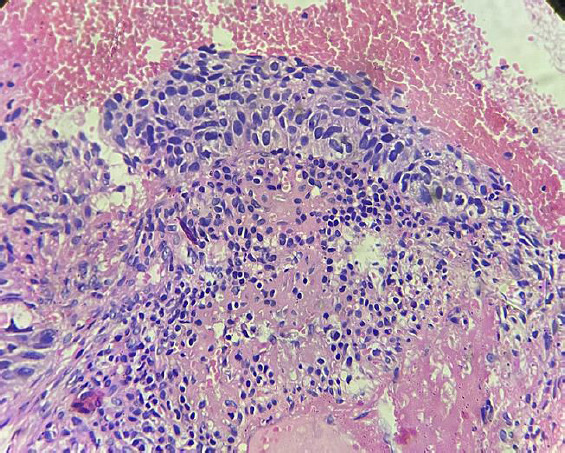
H&E; x 400, high grade dysplasia in ectocervix

**Figure 4:**
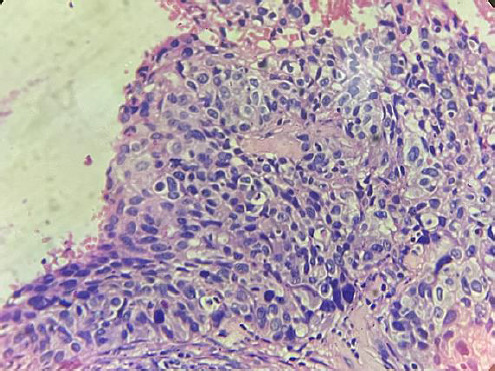
H&E; x 400, squamous cell carcinoma of cervix

**Figure 5:**
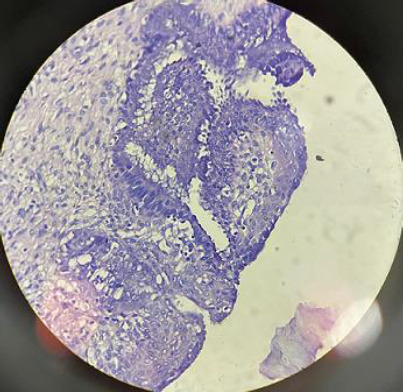
H&E; x 100, squamous metaplasia of cervix

**Figure 6:**
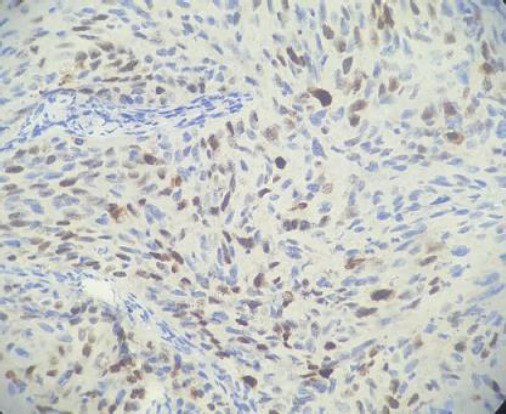
Ki 67 - 70% positivity, x 400, squamous cell carcinoma of cervix

**Figure 7:**
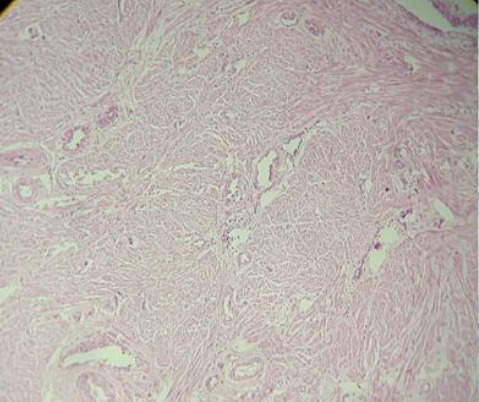
Ki 67 -1-2% positivity, x 100, mild cervical dysplasia of cervix

Of the 140 patients, 38 were diagnosed with mild ectocervical dysplasia. The mean AI increased progressively from the lower to the higher grades of CIN. Apoptotic index significantly increases from CIN 3 to carcinomas. Cell counts also increased notably from CIN I to CIN II and III. A sharp increase in AI from CIN 3 to the carcinoma cervix may suggest potential genetic alterations in CIN3 cells, which could drive the malignant transformation from CIN3 to cervical cancer [1,2]. The flaw in the apoptotic pathway enables cells to multiply despite the presence of genetic mutations. Therefore, AI can be used to evaluate the importance of apoptosis as an indicator of proliferation [3]. Mean AI was calculated using descriptive statistics. Of these, 53 cases had squamous metaplasia, 38 had mild dysplasia (Figure 1), 19 had moderate dysplasia (Figure 2), and 14 had severe dysplasia (Figure 3), 16 cases of malignancy (Figure 4) were evaluated (Table 1). In squamous metaplasia (Figure 5), the mean AI was 1.03, while in mild cervical dysplasia it was 1.51. For moderate cervical dysplasia, the mean AI was 3.06, and for severe dysplasia, the mean AI was 5.23. In cases with overtly malignant cervical lesions, the mean AI was 7.35. The mean AI showed a progressive increase with higher dysplasia grades. The most common age groups for squamous metaplasia (19 cases), mild dysplasia (17 cases), and moderate dysplasia (7 cases) were between 35 and 44 years, whereas for severe dysplasia (5 cases), it was between 55 and 64 years. The most common age group for malignancy (9 cases) was 45-54 years (Table 1). The Ki-67 proliferation index in malignant cases ranged from 70% to 80% (Figure 6), whereas in non-malignant cervical lesions, it was between 1% and 2% (Figure 7). Squamous metaplasia, mild dysplasia, moderate dysplasia, severe dysplasia, and malignancy all showed significant, p-values (Table 2, Table 3). To confirm findings of dysplasia in mild and malignant tumors, Ki-67 staining was performed. Ki-67 expression correlated with the apoptotic index. A higher AI is indicative of high-grade epithelial dysplasia. AI can be utilized as a prognostic marker for the follow-up and monitoring of patients with epithelial dysplasia. AI serves as both a diagnosis and prognosis marker in patients with epithelial dysplasia.

**Table 1:** age wise distribution of dysplasia and malignancies

Age group (in years)	Squamous metaplasia	Mild dysplasia	Moderate dysplasia	Severe dysplasia	Malignancy	Total
25-34	8	11	3	0	0	22
35-44	19	17	7	4	2	49
45-54	10	7	4	2	9	29
55-64	11	3	4	5	4	27
>64	5	0	1	3	1	10
	53	38	19	14	16	140

**Table 2:** grades of cervical epithelial dysplasia, metaplasia, malignancy, with mean apoptosis index and standard deviation

Grades of cervical epithelial dysplasia, metaplasia, and malignancy	Range	Mean AI	Standard deviation
Metaplasia	0.3-1.6	1.03	0.39
Mild dysplasia	1.3-1.7	1.51	0.13
Moderate dysplasia	2.8-3.6	3.06	0.34
Severe dysplasia	4.1- 6.5	5.23	0.88
Malignancy	6.5-8.1	7.35	0.60
AI: apoptosis index			

**Table 3:** mean difference of different grades of cervical dysplasia, metaplasia and malignancy, apoptosis index, p-value

Grades of cervical epithelial dysplasia, metaplasia, and malignancy	Sample size (total-140)	Apoptotic index	p-value
Squamous metaplasia	53	1.03	0.007
Mild dysplasia	38	1.51	< 0.001
Moderate dysplasia	19	3.06	<0.001
Severe dysplasia	14	5.23	<0.001
Malignancy	16	7.35	<0.001

**Statistical analysis:** we used the SocSciStatistics statistics to perform an ANOVA test and post hoc analysis to compare the mean AI across different grades of cervical epithelial dysplasia. A statistically significant difference was found when comparing the mean AI between mild dysplasia, squamous metaplasia, severe dysplasia, and malignancy, with a p-value of < 0.01. The difference in AI between severe and mild dysplasia was 3.72, whereas the difference between mild dysplasia and malignancy was 5.84. In a few cases, the patients exhibited both squamous metaplasia and mild dysplasia. These patients were analysed in the mild dysplasia category to minimize the calculation errors.

## Discussion

This study was conducted in 140 patients with various cervical lesions, and the mean AI was evaluated across increasing grades of lesions, from squamous metaplasia to carcinoma. Our findings showed that the AI significantly increased from squamous metaplasia to malignancy. Similar results were reported in a study by Dey *et al*. [2], where the mean AI increased from lower to higher grades of CIN and carcinoma of the cervix. In premalignant cervical lesions, changes in apoptosis are associated with the onset of invasion. Both apoptosis and cellular proliferation play crucial roles in tumor progression and development. However, the precise interrelationship and specific role of each of these processes in tumor progression remain to be fully elucidated [4,5]. Apoptosis is defined as programmed cell death that occurs without triggering an inflammatory response [6]. Several techniques such as electron microscopy, flow cytometry, gel electrophoresis, immunohistochemistry, and in situ labelling of fragmented DNA are commonly used to detect apoptosis. Apoptosis plays a crucial role in determining the course of potentially malignant cervical disorders. The fourth most frequent malignant tumor of the female genital tract is uterine cervical cancer [7]. Programmed cell death or apoptosis, causes cells to be deleted from both healthy and most likely tumor tissues [8]. The objective of this study was to ascertain the prevalence of apoptotic cells in various premalignant lesion grades and stages. The goal of the current investigation was to determine whether the apoptotic index in the squamous epithelium of cervical cancers and dysplastic cervix correlates [9]. Cell shrinkage, condensed hyperchromatic nodular, crescentic, or beaded nuclear chromatin, and deep eosinophilic cytoplasm are hallmarks of apoptotic cells. All authors have observed that AI is very low in normal cervical epithelium and increases as cervical neoplasia progresses from CIN to carcinoma [5-9]. However, although some women may have a high AI, not all of them develop malignancy, suggesting that additional cofactors are required in the pathogenic pathway between cervical dysplasia [CIN] and carcinoma [10,11].

Ki-67 is a well-known marker for cell proliferation. Its expression is primarily nuclear and observed only in isolated parabasal cells in the normal cervical epithelium. In contrast, Ki-67 expression is stronger and more widespread in CIN and squamous cell carcinoma (SCC) [12,13]. Ki-67 expression was correlated with inflammation (P=0.003) and was more commonly expressed in reactive and atypical lesions than in p16 (INK4a) (P= 0.0080). Human papillomavirus (HPV) type 16 probes stained 54% of cervical neoplastic lesions, and the degree of staining significantly correlated with the severity of neoplasia (P<0.001) [14,15]. The apoptotic cell counts were significantly higher in patients with severe dysplasia than in those with moderate or mild dysplasia. However, there were no significant differences between adenocarcinomas and squamous cell carcinomas. Furthermore, in well or moderately differentiated carcinomas, there was no significant difference in apoptotic cell counts. Notably, the squamous cell carcinoma group showed significantly higher apoptotic cell counts than the preneoplastic lesion group [16]. The role of apoptosis in tumor biology has increased significantly in recent years. The extent of cell turnover and deletion is poorly understood despite the fact that proliferative indices of cancers have long been acknowledged as valuable prognostic markers. The net growth rate of a tumor is influenced by both proliferative activity and the quantity of cells dying through ischemia necrosis or apoptosis. The elimination of cells from both normal and most likely tumor tissues is caused by apoptosis, a type of programmed cell death. Apoptosis is consistently and often observed in adenocarcinomas in situ (AIS), microinvasive adenocarcinomas, and plainly invasive adenocarcinomas. Adenocarcinomas in situ display the highest AI in certain instances [17]. Endocervical glandular cancers frequently exhibit many apoptotic bodies and mitotic figures and are important feature that can facilitate their differentiation from benign and borderline lesions. The apoptotic index was significantly associated with the severity of CIN and not with either age or human papillomavirus infection [18]. Dysregulation in the exfoliation of apoptotic cells and resistance towards apoptosis may be pre-requisites for the pathogenesis of CIN [19].

## Conclusion

This study confirmed the clinical significance of apoptosis in evaluating the course of cervical epithelial dysplasia, which can also serve as a prognostic indicator of premalignant and malignant cervical lesions. To identify apoptosis and understand its function in the mechanism of carcinogenesis, further research with a larger sample size and more sophisticated techniques should be conducted. If medication therapy is available, it will aid in disease prognosis.

### What is known about this topic

- Apoptosis is already regarded as indicator of cell turnover;
- Oral epithelial dysplasia is already considered as preceding evidence for malignancy.

### What this study adds

- The study for cervical dysplasia should be correlated with cervical apoptotic index;
- Study of apoptotic index in cervical epithelium predicts the chances of malignancy;
- It is an easy method to follow patients.
